# Effect of Elevated Neutrophil-to-Lymphocyte Ratio on Adverse Outcomes in Patients With Myocardial Infarction: A Systematic Review and Meta-Analysis

**DOI:** 10.7759/cureus.61647

**Published:** 2024-06-04

**Authors:** Nana O Banahene, Tanya Sinha, Sanam Shaikh, Aung K Zin, Khaldoun Khreis, Sandipkumar S Chaudhari, Calvin R Wei, Sujith K Palleti

**Affiliations:** 1 Internal Medicine, Nanjing Medical University, Nanjing, CHN; 2 Internal Medicine, Tribhuvan University, Kathmandu, NPL; 3 Internal Medicine, Yangtze University, Jingzhou, CHN; 4 Internal Medicine, University of Medicine, Mandalay, MMR; 5 Pediatric, Pécs Medical University, Pécs, HUN; 6 Cardiothoracic Surgery, University of Alabama at Birmingham, Birmingham, USA; 7 Family Medicine, University of North Dakota School of Medicine and Health Sciences, Fargo, USA; 8 Research and Development, Shing Huei Group, Taipei, TWN; 9 Nephrology, Louisiana State University Health Sciences Center, Shreveport, USA

**Keywords:** systematic review and meta analysis, mortality, cardiovascular outcomes, myocardial infarction, elevated neutrophil to lymphocyte ratio

## Abstract

Myocardial infarction (MI), a leading cause of morbidity and mortality globally, is characterized by an underlying inflammatory process driven by atherosclerosis. The neutrophil-to-lymphocyte ratio (NLR), a readily available and cost-effective marker of systemic inflammation, has emerged as a potential predictor of adverse outcomes in patients with MI. This meta-analysis aimed to evaluate the association between elevated NLR and the risk of major adverse cardiovascular events (MACE) and all-cause mortality in patients with MI. A comprehensive literature search was conducted across multiple databases, including Embase, Web of Science, PubMed, and OVID Medicine, to identify relevant studies published from January 1, 2011, onward. Studies reporting the effect of NLR values on MACE and mortality in adult patients with MI, including both ST-elevation (STEMI) and non-ST-elevation (NSTEMI) subtypes, were included. Data extraction and quality assessment were performed independently by multiple authors. The meta-analysis included 37 studies, comprising a total of 18 studies evaluating the risk of MACE and 30 studies assessing all-cause mortality. The pooled analysis revealed a significantly increased risk of MACE (odds ratio [OR] 1.86, 95% confidence interval [CI] 1.53-2.28, *P* < 0.01) and all-cause mortality (OR 2.29, 95% CI 1.94-2.70, *P* < 0.01) in patients with elevated NLR compared to those without elevated NLR. Subgroup analyses stratified by follow-up duration and study design further supported the consistent association between elevated NLR and adverse outcomes. In conclusion, this meta-analysis demonstrates a significant association between elevated NLR and an increased risk of MACE and all-cause mortality in patients with MI. These findings highlight the potential clinical utility of NLR as a prognostic marker and underscore the importance of further research to validate its predictive value and establish optimal cutoff values for risk stratification in this patient population.

## Introduction and background

As a condition characterized by significant morbidity and mortality globally, cardiovascular disease, particularly myocardial infarction (MI), profoundly affects both quality of life and life expectancy for individuals [1]. MI can be divided into two groups: non-ST-elevation MI (NSTEMI) and ST-elevation MI (STEMI) [2]. In recent decades, the management of MI has undergone significant changes due to new approaches and strategies that have fundamentally altered the diagnosis, treatment, understanding, and prevention of this condition [3].

It is well established that atherosclerosis is the underlying pathology of MI, with inflammation playing a crucial role in the progression and onset of atherosclerosis [4]. Considering the link between inflammation and MI, significant effort should be directed toward identifying inflammatory markers associated with MI. One such marker, the neutrophil-to-lymphocyte ratio (NLR), is commonly used and has recently been linked to coronary heart disease [5]. The NLR is calculated by dividing the number of neutrophils by the number of lymphocytes. It has been demonstrated that a higher NLR is linked to poorer outcomes in patients with acute coronary syndromes (ACSs) and established coronary heart disease [6]. Since neutrophil and lymphocyte counts are routinely available in standard blood tests, the NLR can serve as an economical predictor of inflammation and cardiovascular complications [7]. A previous systematic review emphasized the potential use of this accessible and low-cost inflammatory marker for risk stratification in patients with ACSs and those undergoing cardiac revascularization [8].

Previous studies have indicated that the NLR could be an important predictor of short- and long-term mortality in individuals with MI treated with coronary interventions [9-10]. While prior reviews have primarily focused on ACS, the current meta-analysis specifically concentrates on MI, encompassing both STEMI and NSTEMI. Considering the potential significance of NLR in predicting adverse outcomes in patients with MI, we are conducting a pooled analysis of available studies to determine the association between NLR and cardiovascular events, as well as mortality, in this patient population.

## Review

Methodology

We searched for relevant studies from the following databases: Embase, Web of Science, PubMed, and OVID Medicine from January 1, 2011, onwards. The key terms used to search for relevant articles included "neutrophil-to-lymphocyte ratio" and "myocardial infarction," along with their synonyms and medical subject heading (MeSH) terms (Appendix). Additionally, the bibliographic lists of all included articles were manually screened to find more studies relevant to the meta-analysis. We limited the search to studies published in the English language. The search was performed independently by two authors. Disagreements were resolved through consensus and discussion. 

Inclusion/Exclusion Criteria

We included all nonrandomized and randomized studies that reported the effect of NLR values in adult patients with MI, including both STEMI and NSTEMI. We included studies that reported one of the outcomes assessed in this meta-analysis, including MACE and mortality. We excluded studies that included patients other than those with MI. We also excluded case reports, reviews, case series, animal studies, and cross-sectional studies. The study selection was performed by two authors. Initially, titles and abstracts were screened, followed by a detailed screening of the full text. Disagreements were resolved through consensus and discussion. The process of drafting and conducting this systematic review and meta-analysis adhered to the guidelines outlined in the Preferred Reporting Items for Systematic Reviews and Meta-Analyses (PRISMA) statement.

Data Extraction and Quality Assessment

The data extraction process was performed independently by four authors, with two authors extracting data from one half of the included studies, and the other two authors extracting data from the remaining half. In case of any discrepancies in the extracted data values, the respective pairs of authors responsible for each half of the studies resolved the discrepancies cohesively and reconciled the data for their assigned studies. Data extracted from included studies were author name and year, study design, region where the study was conducted, sample size, definition of elevated NLR, follow-up duration, type of MI (STEMI or NSTEMI), and data about the outcomes. Outcomes assessed in this meta-analysis include MACE and mortality. Quality assessment of included studies was conducted using the New Castle Ottawa Scale.

Statistical Analysis

Statistical analysis was conducted using RevMan Version 5.4.1. Results were presented as the combined odds ratio (OR). Random-effect models were employed for all evaluations. The degree of heterogeneity among studies was quantitatively evaluated using the *I*² statistic, with *I*² values categorized as follows: <50% indicating low heterogeneity, between 50% and 75% indicating moderate heterogeneity, and >75% indicating high heterogeneity. All analyses were two-sided, and significance was set at *P*-values < 0.05. 

Results

**Figure 1:**
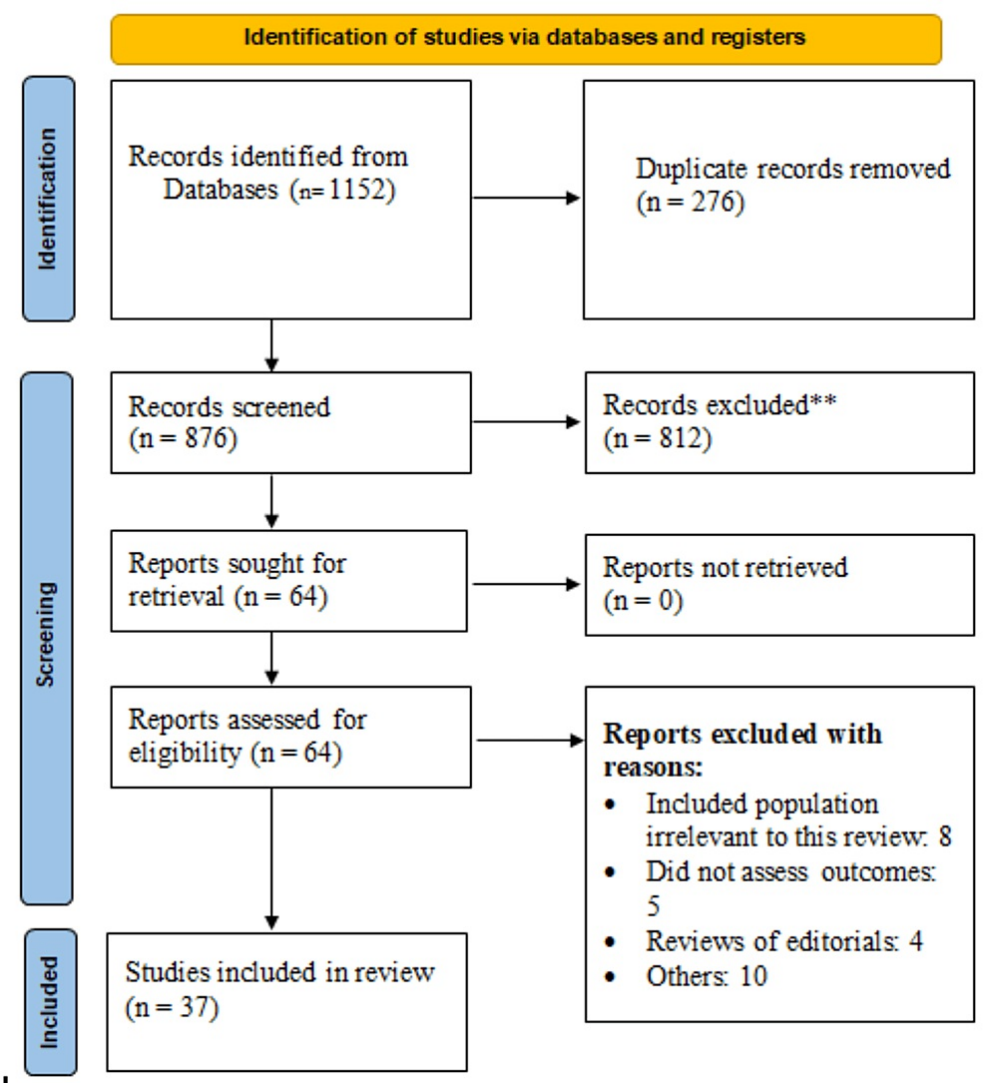
Study selection process (PRISMA flowchart). PRISMA< Preferred Reporting Items for Systematic Reviews and Meta-Analyses

Figure 1 shows the study identification and screening process in the PRISMA flowchart. A thorough search of databases and registries for relevant literature yielded 1,152 possible papers. After eliminating 276 duplicates, 876 studies remained for abstract screening. After screening the abstracts, 812 studies were eliminated. Sixty-four studies were still awaiting full-text review. Ultimately, this meta-analysis included 37 papers. Table 1 presents the characteristics of the included studies. Of the 37 included studies, 17 were prospective and 20 were retrospective. The sample sizes of the studies ranged from 107 to 2,618 patients. The majority of the studies were conducted in China (29%), Turkey (29%), and Korea (14%). Table 2 presents the quality assessment of the included studies.

**Table 1:** Characteristics of included studies. NR, not reported

Author Name	Year	Region	Study design	Sample size	Follow-up duration	Criteria of high NLR
Akpek et al [9]	2012	Turkey	Retrospective	418	In hospital	>3.3
Arbel et al. [10]	2014	Israel	Prospective	538	72 Months	>6.5
Chen et al. [11]	2020	China	Retrospective	107	24 Months	NR
Chen et al. [12]	2023	China	Retrospective	1550	In hospital	NR
Ergelen et al. [13]	2014	Turkey	Retrospective	2410	21 Months	<6.97
Gazi et al. [14]	2015	Turkey	Retrospective	522	In hospital	<5.77
Gul et al. [15]	2014	Turkey	Prospective	308	In hospital	<3.04
Gürdal et al. [16]	2020	Turkey	Retrospective	320	21 Months	NR
Han et al. [17]	2013	Korea	Prospective	326	12 Months	<3.30
Hartopo et al. [18]	2015	Indonesia	Prospective	165	In hospital	>6.2
He et al. [19]	2014	China	Prospective	692	21 Months	>3.16
Her et al. [20]	2017	Korea	Retrospective	172	41 Months	>5.8
Hong et al. [21]	2019	Korea	Retrospective	309	24 Months	<3.88
Jadhav et al. [22]	2022	India	Prospective	400	In hospital	>5.20
Ji et al. [23]	2021	China	Retrospective	2618	In hospital	>5.509
Júnior et al. [24]	2018	Brazil	Prospective	466	In hospital	>3.7
Karaca et al. [25]	2024	Turkey	Retrospective	641	In hospital	>5.95
Konishi et al. [26]	2017	Japan	Retrospective	331	12 Months	NR
Li et al. [27]	2020	China	Prospective	502	In hospital	NR
Lin et al. [28]	2021	China	Retrospective	181	24 Months	>8.16
Machado et al. [29]	2019	Brazil	Prospective	644	1 Month	<9.45
Oh et al. [30]	2020	Korea	Retrospective	1057	12 Months	>4.3
Pan et al. [31]	2015	China	Prospective	636	In hospital	>3.0
Park et al. [32]	2013	Korea	Prospective	325	36 Months	<5.44
Park et al. [33]	2018	Korea	Prospective	326	68 Months	NR
Sasmita et al. [34]	2021	China	Retrospective	217	In hospital	<7.3
Sawant et al. [35]	2014	United States	Retrospective	250	12 Months	>7.4
Sen et al. [36]	2013	Turkey	Prospective	204	36 Months	<4.27
Sharma et al. [37]	2023	India	Prospective	110	In hospital	>3.0
Sigirci et al. [38]	2021	Turkey	Retrospective	1186	In hospital	NR
Tanriverdi et al. [39]	2017	Turkey	Retrospective	368	In hospital	>5.47
Tavares et al. [40]	2022	Brazil	Retrospective	1860	In hospital	<4.0
Wang et al. [41]	2022	China	Retrospective	855	In hospital	NR
Wang et al. [42]	2023	China	Retrospective	782	12 Months	<4.8
Zhang et al. [43]	2023	China	Prospective	990	28 Months	NR
Zhang et al. [44]	2015	China	Prospective	248	12 Months	NR
Zuin et al. [45]	2017	Italy	Prospective	2341	12 Months	>3.9

**Table 2:** Quality assessment of included studies.

Author name	Selection	Comparison	Assessment	Overall quality
Akpek et al. [9]	4	2	2	Good
Arbel et al. [10]	3	2	3	Good
Chen et al. [11]	4	2	3	Good
Chen et al. [12]	4	2	2	Good
Ergelen et al. [13]	4	2	2	Good
Gazi et al. [14]	4	1	3	Good
Gul et al. [15]	3	2	3	Good
Gürdal et al. [16]	3	1	3	Good
Han et al. [17]	3	1	3	Good
Hartopo et al. [18]	2	2	3	Good
He et al. [19]	2	1	3	Fair
Her et al. [20]	2	2	3	Good
Hong et al. [21]	3	2	2	Good
Jadhav et al. [22]	3	2	2	Good
Ji et al. [23]	4	2	3	Good
Júnior et al. [24]	3	2	2	Good
Karaca et al. [25]	4	2	3	Good
Konishi et al. [26]	4	2	2	Good
Li et al. [27]	3	2	3	Good
Lin et al. [28]	3	1	2	Fair
Machado et al. [29]	3	2	3	Good
Oh et al. [30]	3	2	3	Good
Pan et al. [31]	2	1	2	Fair
Park et al. [32]	2	1	3	Fair
Park et al. [33]	3	1	2	Fair
Sasmita et al. [34]	3	2	2	Good
Sawant et al. [35]	3	2	2	Good
Sen et al. [36]	3	2	2	Good
Sharma et al. [37]	4	2	3	Good
Sigirci et al. [38]	3	2	2	Good
Tanriverdi et al. [39]	3	2	3	Good
Tavares et al. [40]	3	2	2	Good
Wang et al. [41]	3	1	2	Fair
Wang et al. [42]	3	1	2	Fair
Zhang et al. [43]	4	2	3	Good
Zhang et al. [44]	3	2	2	Good
Zuin et al. [45]	3	2	2	Good

NLR and MACE Rate 

**Figure 2:**
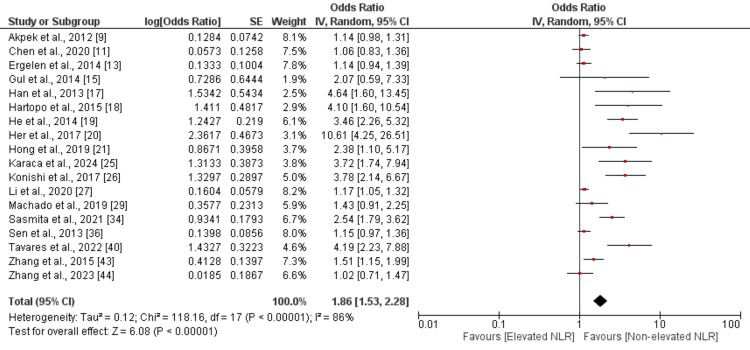
Forest plot evaluating the association between high NLR and MACE. Sources: [9,11,13,15,17-21,25-27,29,34,36,40,43-44]. NLR, neutrophil-to-lymphocyte ratio; MACE, major adverse cardiovascular event; CI, confidence interval; SE, standard error

Figure 2 presents the results of 18 research that investigated the risk of MACE. A pooled analysis of eighteen studies revealed that individuals with elevated NLR had a 1.86-fold increased risk of MACE compared to those without elevated NLR. This difference was statistically significant (OR 1.86, 95% confidence interval [CI] 1.53 - 2.28, *P* < 0.01). The study's results showed a high degree of heterogeneity (*I*^2^ = 86%).

NLR and All-Cause Mortality 

**Figure 3:**
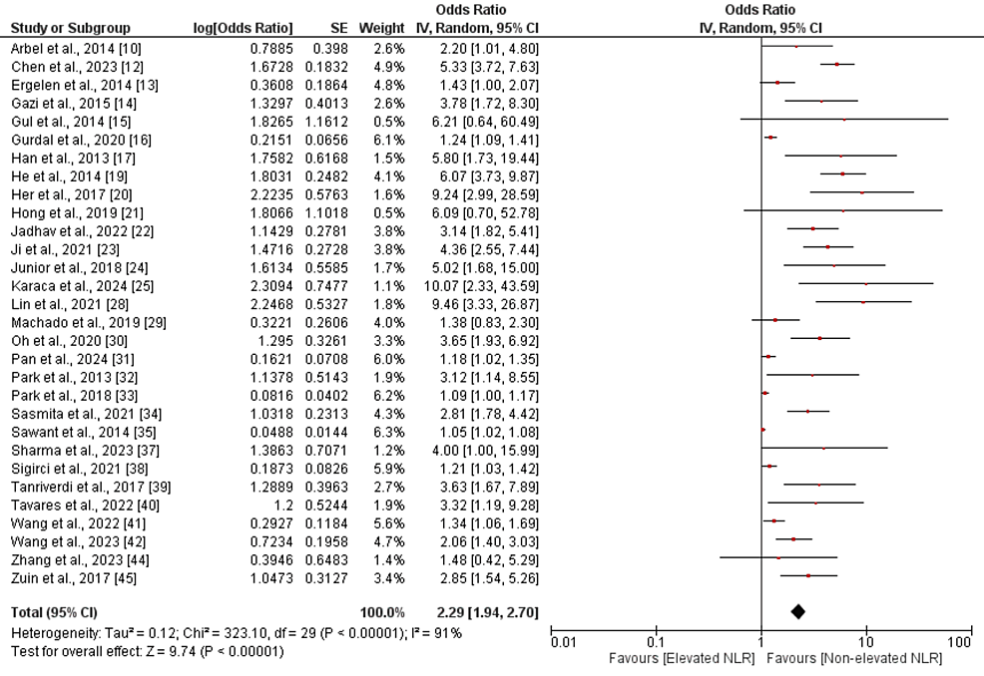
Forest plot evaluating the association between high NLR and all-cause mortality. Sources: [10,12-17,19-25,28-35,37-42,44-45]. NLR, neutrophil-to-lymphocyte ratio; CI, confidence interval

Thirty studies evaluated the risk of all-cause death; the findings are shown in Figure 3. A combined evaluation of 30 studies revealed that individuals with elevated NLR had an all-cause mortality risk 2.29 times greater than those without elevated NLR, and this difference was statistically significant (OR 2.29, 95% CI 1.94-2.70, *P*-value < 0.01). The study's results showed a high degree of heterogeneity (*I*^2^ = 91%). 

*Subgroup Analysis* 

Table 3 presents a meta-analysis of studies evaluating the association between elevated NLR and two clinical outcomes: all-cause mortality and major adverse cardiovascular events (MACEs). For all-cause mortality, studies with a follow-up duration of less than one year showed a higher OR of 2.65 (95% CI 1.92-3.67) for elevated NLR compared to non-elevated NLR, while studies with a follow-up of one year or more had a lower OR of 2.01 (95% CI 1.64-2.47). Both prospective (OR 2.40, 95% CI 1.75-3.30) and retrospective (OR 2.45, 95% CI 1.90-3.16) study designs consistently demonstrated an increased risk of all-cause mortality with elevated NLR. For MACE, studies with a shorter follow-up duration (less than one year) had a higher OR of 2.32 (95% CI 1.44-3.74), while those with longer follow-up (one year or more) had a lower OR of 1.70 (95% CI 1.34-2.17). Retrospective studies showed a higher OR of 2.25 (95% CI 1.54-3.29) for MACE compared to prospective studies (OR 1.64, 95% CI 1.29-2.09). This shows that irrespective of the duration. NLR can be used as a potential biomarker to predict adverse events in patients with MI.

**Table 3:** Results of subgroup analysis. OR, odds ratio; CI, confidence interval

Outcomes	Subgroups	Categories	Number of studies	OR (95% CI)
All-cause mortality	Follow-up duration	Less than one year	15	2.65 (1.92-3.67)
		One year or more	15	2.01 (1.64-2.47)
	Study design	Prospective	14	2.40 (1.75-3.30)
		Retrospective	16	2.45 (1.90-3.16)
Major adverse cardiovascular events	Follow-up duration	Less than one year	7	2.32 (1.44-3.74)
		One year or more	11	1.70 (1.34-2.17)
	Study design	Prospective	10	1.64 (1.29-2.09)
		Retrospective	8	2.25 (1.54-3.29)

Discussion

According to our meta-analysis of 37 studies, patients with elevated NLR had a considerably greater risk of MACE and mortality than their peers. Based on the results of this meta-analysis and the NLR index's widespread availability and affordability, our research suggests that NLR has a promising prognostic marker for patients with MI.

Our results are consistent with those of other meta-analyses conducted to date. A meta-analysis conducted in 2024 that comprised 90 publications and 45,990 participants found that the mean NLR was significantly higher in ACS patients with MACE compared to those without MACE [46]. A 2018 meta-analysis, which encompassed 14 studies and a total of 10,245 patients who underwent PCI following STEMI, revealed significant associations between elevated NLR and various adverse outcomes. These included increased mortality, higher rates of MACE, an elevated risk of stent thrombosis, and poorer long-term survival outcomes [47].

Elevated NLR levels have been consistently associated with an increased risk of adverse cardiovascular outcomes following MI. This relationship stems from the intricate interplay between neutrophils and lymphocytes in the inflammatory response post-MI [48]. Neutrophils contribute to the acute inflammatory phase, while lymphocytes play a crucial role in the resolution of inflammation and tissue repair. An imbalance in this ratio reflects the dysregulation of the immune response, which is a hallmark of atherosclerosis and subsequent cardiovascular events [49]. Furthermore, NLR serves as a readily available and cost-effective biomarker, making it particularly attractive for risk stratification in clinical practice. Its simplicity, coupled with its prognostic significance, underscores its potential as a valuable tool for identifying patients with high-risk MI who may benefit from intensified therapeutic interventions and closer monitoring to mitigate the risk of MACE [50]. Overall, the NLR holds promise as a clinically relevant biomarker for predicting MACE in individuals with MI, offering valuable insights into the pathophysiology of cardiovascular disease and opportunities for optimizing patient management strategies. In contrast to alternative biomarkers of the systemic inflammatory response, acquiring the NLR is simpler, requiring only a basic blood count [51]. Additionally, compared to other leukocyte subtypes, it demonstrates greater stability [23]. Consequently, the NLR presents itself as a practical and economically viable substitute for other biomarkers. Nevertheless, as with many biomarkers, further studies are necessary to ascertain the genuine prognostic significance of its cutoff point. NLR may also be added to other potential scores or predictors, such as the thrombolysis in MI (TIMI) and Global Registry of Acute Coronary Events (GRACEs) risk scores, to better define patients' short- and long-term prognoses. This could be especially important for patients with NSTEMI, for whom early risk classification could help determine when to perform revascularization [52-53]. It is important to note that in patients with NSTEMI, the NLR may be altered due to coexisting comorbidities such as diabetes, COVID-19, other inflammatory syndromes, or concurrent infections, which could affect the interpretation and prognostic value of NLR in this population. In terms of the current meta-analysis, studies included excluded patients with comorbidities in which NLR is altered.

Considering the aforementioned points, the predictive capabilities of NLR concerning the risk of MACE appear to hold the greatest clinical significance. The challenge of identifying patients with the highest susceptibility to developing MACE within a foreseeable and well-defined timeframe remains considerable in clinical practice. Recognizing individuals at risk of MACE could potentially lead to more tailored and optimized care for this specific patient population. Further investigation, particularly to validate the predictive efficacy of NLR in predicting MACE, is warranted. Additionally, research should aim to determine the optimal timeframe for NLR-based prediction accuracy and specify which outcomes, variably delineated across studies as components of the composite MACE endpoint, are most effectively predicted by NLR's predictive attributes.

Study Limitations

Our research has several potential limitations. We cannot overlook potential differences in baseline characteristics and contemporaneous risk variables across patients with varying NLR levels since we conducted a study-level meta-analysis. Additionally, there are variations in the inclusion and exclusion criteria among the included studies, along with discrepancies in defining high and low NLR levels. In general, we cannot dismiss the computed heterogeneity between the research, as indicated by the *I*^2^ statistic. Therefore, combining data from several studies might not be suitable. Nevertheless, all analyses were conducted using the random-effects model, which mitigates some of the between-study variances. Furthermore, the majority of studies reported a statistically significant positive correlation between NLR and endpoints, with the degree of association appearing somewhat heterogeneous across studies. Future research should focus on validating NLR's predictive efficacy for MACE in diverse populations, determining optimal cutoff points, and integrating NLR with existing risk scores like TIMI and GRACE. Additionally, studies should establish the best timeframe for NLR-based predictions and specify which MACE components are most effectively predicted.

## Conclusions

In conclusion, this meta-analysis demonstrates that an elevated NLR is significantly associated with an increased risk of MACE and all-cause mortality in patients with MI. The NLR is a readily available and cost-effective biomarker that holds promising potential for risk stratification and prognostic assessment in patients with MI. Incorporating NLR into existing risk prediction models could enhance the identification of high-risk individuals, enabling more targeted and optimized management strategies. However, further research is warranted to validate the predictive efficacy of NLR and establish optimal cutoff values for clinical decision-making.

## Appendices

Appendix

Search Strategy for PubMed

(("Neutrophils"[Mesh] OR neutrophil*[tiab] OR "Leukocyte Count"[Mesh]) AND ("Lymphocytes"[Mesh] OR lymphocyte*[tiab]) AND (ratio[tiab] OR "Ratio"[Mesh])) AND ("Myocardial Infarction"[Mesh] OR "myocardial infarction"[tiab] OR "heart attack"[tiab]  OR STEMI[tiab] OR NSTEMI[tiab]) AND ("Mortality"[Mesh] OR mortality[tiab] OR death[tiab] OR "Major Adverse Cardiovascular Events"[Mesh] OR MACE[tiab] OR "cardiovascular event"[tiab])
